# Visualizing cancer and survivorship with generative AI?—an exploration of breast, prostate, and pancreatic cancer imagery

**DOI:** 10.1007/s11764-025-01843-z

**Published:** 2025-06-07

**Authors:** Miguel Varela-Rodríguez, Stefanie Plage

**Affiliations:** 1 Department of Sociology and Social Work, Faculty of Commerce, University of Valladolid, Pl. Campus Universitario, 1, 47011 Valladolid, Spain; 2 School of Social Science, Faculty of Humanities, Arts and Social Sciences, The University of Queensland, Brisbane, Australia; 3 Australian Research Council Centre of Excellence for Children and Families Over the Life Course, Brisbane, Australia

**Keywords:** Visual communication, Generative AI, Advocacy, Breast cancer, Prostate cancer, Pancreatic cancer

## Abstract

**Purpose:**

Generative Artificial Intelligence (GAI) is transforming visual communication in the context of cancer survivorship, presenting opportunities to innovate advocacy while also posing risks for social representation. This study explores how GAI visualizes cancer and survivorship, focusing on its ability to reflect diverse experiences and its limitations.

**Methods:**

We analyzed 262 images generated by Dall-E and Stable Diffusion using prompts related to breast, prostate, and pancreatic cancer. A mixed-methods approach examines how GAI utilizes cancer signifiers, visualizes the impact of cancer on individuals, and represents people with cancer.

**Results:**

GAI frequently reproduces cancer tropes, such as prescriptive positivity, and fails to depict medical treatments or embodied experiences unless explicitly prompted. AI-generated images predominantly featured White, female subjects, particularly in breast cancer contexts, reflecting broader biases in public discourse. While GAI tools can produce inclusive visuals, achieving this requires users to have nuanced knowledge of cancer and survivorship, limiting accessibility for lay GAI users.

**Conclusions:**

GAI can support cancer communication but risks perpetuating stereotypes and excluding less visible experiences of cancer. Our findings offer practical insights to support the design of advocacy materials and campaigns, particularly through improved prompt literacy and inclusive image generation strategies.

**Implications for Cancer Survivors:**

Inclusive and respectful visual representation is critical for capturing the diverse realities of cancer survivorship, which in turn affects the wellbeing of cancer survivors and carers. Collaborative efforts among researchers, advocates, and GAI developers are necessary to improve datasets and foster accessible tools, ensuring that GAI supports rather than undermines cancer survivorship advocacy.

**Supplementary Information:**

The online version contains supplementary material available at https://doi.org/10.1007/s11764-025-01843-z.

## Introduction

Generative artificial intelligence (GAI) is transforming advocacy by offering new ways to represent cancer survivorship. For decades, survivorship has been central to cancer communication, shaping public perceptions through stories of resilience, recovery, and community, and, in turn, affecting the identity and wellbeing of people with cancer. This discourse has also created a standardized image of battle and triumph that omits the medical reality of the disease and often excludes those whose experiences do not align with it or who die with cancer. Since the 1970 s, advocates have worked to broaden the public image of cancer, incorporating experiences beyond dominant survivorship imagery.

Today, tools like ChatGPT and Stable Diffusion can produce realistic images to support cancer research and advocacy, and AI-generated images now appear in image banks under the keyword “cancer”. But what do AI-generated images of cancer and survivorship actually look like? This study analyzes 262 images generated by Dall-E 2.0 and Stable Diffusion^1^ using cancer-related prompts, with a focus on breast, prostate, and pancreatic cancer.

We explore how AI “imagines” cancer treatment, visualizes cancer advocacy, and envisions life with and beyond cancer. Our aim is to understand how these tools might impact cancer communication and shape public perception, aligning with the broader remit of cancer survivorship to connect clinical practice, public policy, and evidence in ways that improve the experience of those affected by cancer [2].

While prior research has extensively explored cancer representation in media, particularly in relation to the discourse of survivorship [3–5], few studies have examined how emerging technologies like GAI might reshape these narratives. A recent paper by Senft Everson et al. [6] takes an important first step by comparing AI-generated images produced with the prompts “cancer survivor” and “cancer patient,” highlighting how prompt language affects tone and representation. However, other existing work still largely focuses on content generated by users (e.g., Instagram, blogs, campaigns), or on media created by humans [7–10]. Given the rapid growth, standardization, and increasing accessibility of GAI platforms, further analysis is needed to understand how these tools visualize illness across a broader range of prompts, identities, and cancer types and how they may replicate or subvert cultural biases.

This study contributes to this scholarship by analyzing visual outputs from two leading GAI platforms, evaluating how they render cancer across diverse prompts. In doing so, we offer new insights into how automated image generation may extend existing visual discourses around survivorship.

To capture a spectrum of visibility and cultural representation, we focused on three cancer sites: breast, prostate, and pancreas. Breast cancer was chosen due to its prominent public profile and well-established visual culture, shaped by decades of survivorship narratives and advocacy campaigns. Prostate cancer, while common, remains less publicly visible and is rarely centered in visual advocacy, offering a useful counterpoint to breast cancer’s iconography. Pancreatic cancer, meanwhile, represents a highly lethal and underrepresented cancer site, with minimal advocacy infrastructure and few culturally recognizable symbols. Together, these sites allow us to investigate how GAI engages with cancers that vary in gender association, survivorship framing, and public awareness—revealing how visibility and discourse shape AI-generated imagery.

We aim to help social workers, advocacy groups, and medical practitioners harness GAI’s potential while mitigating the risks of reinforcing stereotypes or marginalizing underrepresented groups.

Beginning with an introduction to visual discourses on cancer and social issues around AI, we then provide a detailed description of our method for generating and interpreting images. Our results focus on three areas: cancer signifiers, embodied manifestations of cancer, and representations of individuals who experience cancer. Throughout, we highlight differences between the two GAI tools we used. Finally, we discuss the practical implications for public advocacy and cancer communication and suggest avenues for future research.

### The visual discourse on cancer in online media

From the 1950 s, survivorship discourse emerged to break with the grim image of medical photography and cancer representation. Where earlier images focused on tumors and pictured the patient solely through the medical gaze, survivorship emphasized recovery, created spaces for sharing, and normalized the illness and “fight” against it [4, 11]. Survivorship quickly became central to cancer communication, sometimes casting patients as champions or heroes [12, 13], and often aligning with ideals of community, responsibility, and—in the case of breast cancer survivorship—traditional femininity [3].

By the 1960 s, campaigns embraced a rhetoric of battle [3, 4, 14–16], adopting the ribbon and the color pink as symbols [17] and portraying people with breast cancer as responsible, strong, and triumphant, ready to “restore their femininity.” Survivorship imagery came to dominate the visual discourse on cancer [5, 17, 18] and became ubiquitous in consumer and specialized magazines [19, 20] and, later, in social media [7, 21–23]. The image of young, White women, surrounded by pink soon spread over to other cancer sites, in a process that Bell calls “breast-cancer-ization” [5].

Despite criticism for promoting an idealized standard that fails to reflect the diversity of cancer experiences [5, 24–26], the visual discourse of survivorship was reinforced in the early 2000 s and 2010 s by social media algorithms, cultural norms, and the pursuit of “likes”. Although some cancer photography in online media offers a critical view [27, 28], many users adjust their posts to fit into the “social media economy,” where visual symbols of survivorship are a valuable currency [10]. Businesses, celebrities, and the public frequently display solidarity through pink ribbons and survivorship language or by modeling the “right” attitudes for patients [29–32].

Advocacy groups, patients, and carers have worked to create a more representative image of cancer, incrementally broadening the depiction of experiences [8, 9, 27]. While still at an early stage, artificial intelligence has also proven useful here. In 2023, the Metastatic Breast Cancer Organization (AECMM) in Spain used GAI to generate art inspired by patients’ stories, which was then displayed in hospitals [33]. In 2024, Breast Cancer Now in the UK employed GAI to create images depicting cancer patients at future events they might not live to experience, reflecting the fear of death and underscoring the need for awareness [34]. Meanwhile, image stock sites like Shutterstock have also added AI-generated images to their “cancer advocacy” and “cancer support” collections.

### An emerging social science of generative AI

Generative AI refers to computer programs that generate “seemingly new, meaningful content” [35] based on user-provided parameters or “prompts.” Since 2017, GAI has grown exponentially, becoming a leading technology at the consumer level [36]. GAIs initially learn from a large pool of examples, sometimes sourced from Google or from social media, and incorporate user feedback to improve their results. This process is increasingly efficient, raising the possibility that GAI may even surpass human capabilities in some areas [37], including image creation. Among academics, this rapid growth has spurred numerous publications since 2019 [38], with a new social science field attending to its implications and challenges.

Concerns around GAI include warnings of catastrophic risks, even “extinction” without proper regulation [39]: threats to privacy, the spread of mis/disinformation, the creation of malicious content [40], and the issue of “deepfakes” are well-known issues [41–43]. Extant work has also discussed the risk of bias and how GAIs may reproduce non-inclusive or harmful discourses [44]. For example, GAIs appear to lean on gender stereotypes, portraying women as shyer and more approachable than men [45] or depicting them in traditionally gendered jobs, such as nursing or education. These platforms also introduce ethnic biases, particularly when users write unspecific prompts [46–50], and reflect the social biases of coders and from databases, including social media [44, 51, 52].

In healthcare, generative systems have been shown to replicate demographic biases, raising concerns about the representational fairness of AI-generated medical imagery [53]. These reflect broader findings in visual artificial intelligence, where biases in training data, labelling, and architecture have been documented [54]. Specifically on AI-generated images of cancer, Senft Everson et al. [6] find that prompts using “survivor” tend to produce optimistic, de-medicalized imagery, while “patient” prompts yield more somber and clinical depictions. Their study highlights how prompt language can influence the tone and framing of AI-generated cancer imagery.

Our study broadens the scope of inquiry by analyzing a wider, systematically varied set of prompts across three cancer sites—breast, prostate, and pancreas. Employing visual content analysis, we explore how GAI reflects, reinforces, or challenges dominant survivorship discourse. Based on our denotative and connotative analyses of 262 images, we discuss the implications of using AI-generated imagery for public perceptions of cancer and survivorship.

## Methodology

We conducted an exploratory study on AI-generated images, guided by three research questions:

- How do GAIs signify the presence of cancer in their images?
- How does cancer affect the body according to AI images?
- What do people who experience cancer look like, and how do they feel and behave in AI-generated images?

To address these questions, we created 33 prompts with increasing levels of complexity (see Online Resource 1). We began with simple, single-word terms like “cancer” and gradually introduced two types of modifiers to reflect greater specificity with respect to cancer site or identity constructs. This structure allowed us to observe how GAIs respond to progressively more detailed language and common survivorship terms. All prompts were intended to simulate the perspective of a layperson seeking to produce images for cancer advocacy, using simple, nontechnical language. As such, this study does not engage with GAI architecture or prompt engineering frameworks.

We prompted the two GAIs to generate images related to three cancer sites: breast, prostate, and pancreas. These sites were selected to reflect a spectrum of public visibility, gender association, and advocacy infrastructure. This diversity allowed us to examine how GAI responds to both dominant visual discourse and less represented forms of illness. Each cancer type was then paired with prompt modifiers to explore how the platforms depict treatment, identity, and emotional tone across the results.

### Image production, inclusion, and download

We queried the GAIs Dall-E 2.0^2^ and Stable Diffusion using the prompts defined. Each GAI generated up to four different images for each prompt. Two duplicate images were obtained and removed from the data set. The remaining 262 images were downloaded to a shared drive for analysis.

### Image annotation

Images were annotated in Microsoft Excel using emerging categories drawn from social semiotic and visual discourse analysis. This method begins with an initial round of open-ended observation, where elements that stand out visually or thematically—such as gestures, colors, symbols, or mood—are annotated without a pre-set coding scheme. These annotations are then clustered and refined into a structured framework for analysis. Following Rose [55], Kress and van Leeuwen [56], and Rodríguez and Dimitrova [57], we developed codes for visual elements such as mood (e.g., smiling, crying, frowning), ethnic cues (e.g., skin tone, facial features, cultural signs), treatment indicators (e.g., headbands, IV lines, hospital settings), and symbolic signifiers (e.g., pink ribbons, colors), based on established survivorship literature [6, 13, 58, 59]. Mood was coded by assessing facial expressions and visible actions, following Feng and Halloran [60], while ethnic cues were annotated based on similar work by Park et al. [23].

The final set includes 38 codes and 8 clusters addressing cancer signifiers, gender, age, skin tone, number of people in the image, body features, indicators of cancer treatment, and emotions (see Online Resource 2). Both authors independently annotated all images, resolving discrepancies (38 in total) through discussion.

Although qualitative analysis software was considered, Microsoft Excel was chosen for this content analysis for its flexibility and efficiency in managing variables and facilitating side-by-side annotation. Given the exploratory and descriptive nature of the study, the analysis is qualitative and interpretive, rather than statistical.

All images used in this paper were generated via artificial intelligence and do not contain any real people. While the study did not involve human participants or real user data necessitating informed consent, we considered the ethical implications of our research at every step. For example, we were acutely aware of our own positionality as qualitative researchers with extensive knowledge of the cultural aspects of cancer survivorship and how this shaped our analyses.

## Results

A total of 262 images were obtained: 56 for breast cancer, 39 for pancreatic cancer, and 47 for prostate cancer (see Online Resource 3). Our analysis revealed three primary ways in which GAIs visualize cancer: indicating its presence through signifiers, illustrating its physical impact, and representing individuals affected by it. Table 1 provides an overview of the key patterns observed across the image sets. We further discuss the main analytical foci below.

**Table 1:** Summary of results

Results theme	Key findings	Implications
3.1 Signifiers of cancer	Cancer is most often signified through pink, ribbons, and color saturation. Breast cancer symbolism dominates even generic prompts. Signifiers appear more with identity- or awareness-related terms	GAI draws heavily on existing awareness campaign aesthetics, particularly those associated with breast cancer. Without guidance, outputs may reinforce symbolic norms that obscure clinical realities or non-dominant cancer narratives
3.2 Depictions of treatment and impact on the body	Treatment indicators like headbands, hair loss, or medical settings appear in fewer than half of the images. Medical elements are shown only in response to prompts including “patient” or “person with cancer.”	Visual language defaults to symbolism rather than medical realism, often decontextualizing cancer. Reliance on stylized or sanitized imagery risks underrepresenting the embodied and clinical aspects of cancer unless specifically prompted
3.3.1 Appearance	Most people shown are women, often White and adult. Ethnic diversity is low unless specifically prompted. Gendered cues are common but not universal; some androgynous figures appear	Reflects and amplifies biases found in traditional media. Without prompt specificity, GAI outputs may exclude marginalized identities and reinforce a narrow visual template for illness and survivorship
3.3.2 Emotion	GAIs depict a mix of positive and negative emotions, often exaggerated or ambiguous. Facial expressions sometimes contradict body language. Desaturation, gesture, and accessories shape emotional tone	Image mood is influenced by both prompt and aesthetic norms. Some images appear stylized to match social media formats. This may distort the affective realities of illness and create challenges for empathetic or accurate advocacy communication
Differences by platform	**Stable diffusion:** Rarely showed medical settings or equipment. Depicted greater emotional range, including sadness or distress, often using desaturated color palettes. Symbolism was sometimes distorted or surreal (e.g., ribbons emerging from skin, obscuring faces)	**Dall-E 2.0:** More frequently depicted hospital beds, IVs, and medical gowns, especially in response to “patient” prompts. “Survivor” and “awareness” prompts produced stylized, polished images with bright colors and coordinated outfits, leaning on breast cancer survivorship. Emotions were often positive or neutral

### Signifiers of cancer

While visual metaphors such as “invader” or “strange mass” are common in written and verbal cancer communication, representing cancer visually is complex. Without showing its physical impact, images rely on signifiers like icons, text, or symbols (see Online Resource 4).

In our sample, 212 images (80.9%) used at least one signifier of cancer, such as a specific color or a ribbon. Pink was the most common signifier (44% of all images), followed by blue (17.6%) and purple (15.6%), each linked to specific cancer types (pink for breast, blue for prostate, purple for pancreas). After pink, the ribbon is the most common signifier (31.3% of all images). Images also used headbands or hair loss, signs of treatment that we discuss in the next section.

Prompting for a specific cancer site increased the use of signifiers: while 40.8% of non-site-specific images used signifiers, the percentage rose for “pancreatic cancer” (56.4%), for “prostate cancer” (68%), and for “breast cancer” (84%). We also observed differences in the use of signifiers when introducing identity modifiers. Notably, 94.6% of “awareness” images used signifiers, mostly pink and the ribbon.^3^ Similarly, combining “survivor” and “awareness” or prompting for a “person with cancer” resulted in high percentages of signifiers (87.5% and 86.6%, respectively). “Survivor” images used signifiers 73.2% of the time, whereas “patient” images did so 67.1% of the time.

**Fig. 1:**
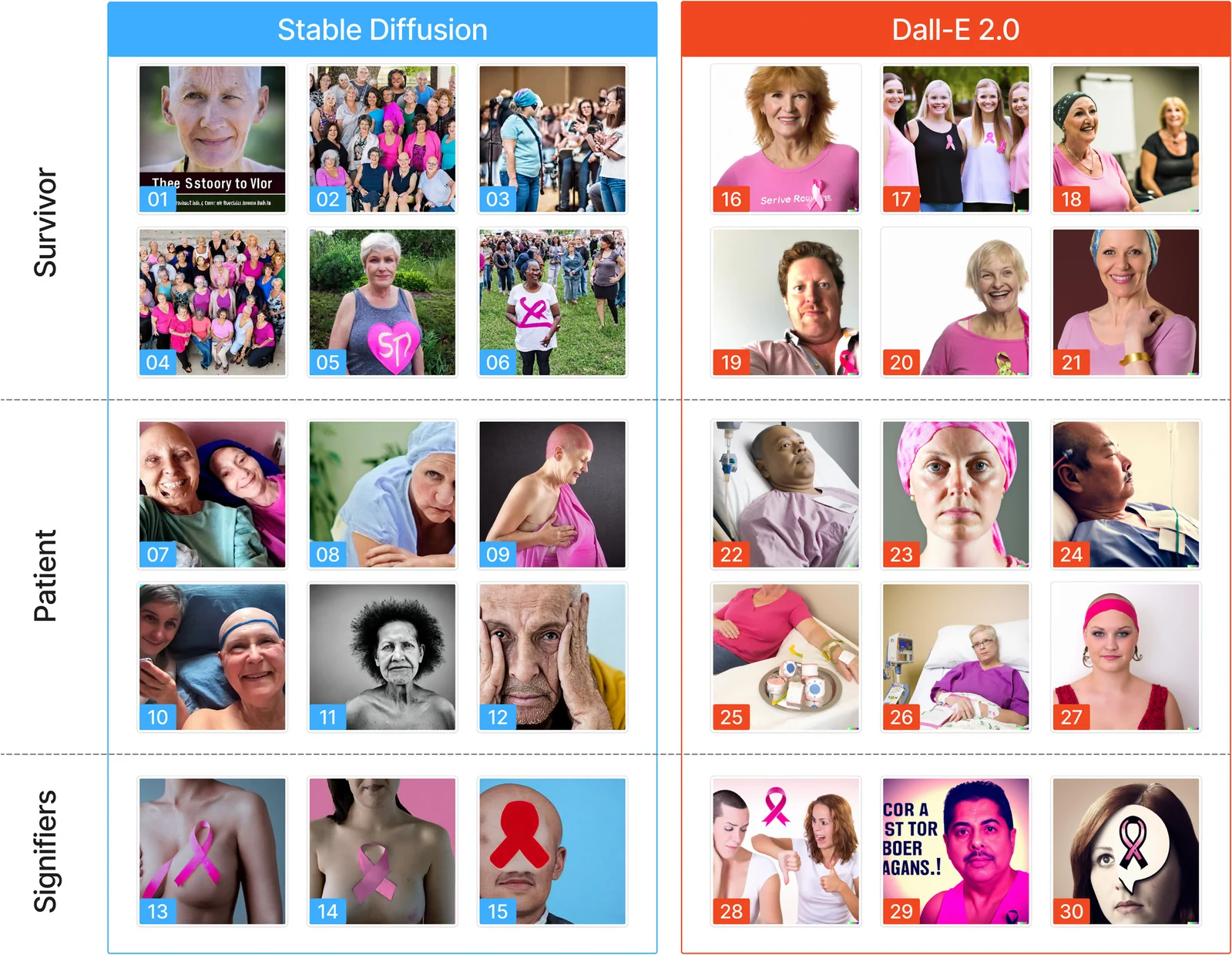
Examples of AI-generated imagery across survivor, patient, and signifier representations in cancer-related prompts. Images were selected to illustrate the results. The figure contrasts outputs from Stable Diffusion (left) and DALL-E 2.0 (right), organized by prompt type. “Survivor” images depict smiling, socially integrated individuals, often styled with pink clothing or symbolic elements. “Patient” images present more clinical, isolated, or somber depictions, sometimes including hospital settings and visible treatment effects. The “Signifiers” row illustrates how each platform uses visual symbols—particularly pink ribbons

The two GAIs used signifiers differently. When depicting “survivors,” Dall-E shows patients wearing bright pink makeup, or groups of young, healthy women in matching clothes (see Fig. 1, images 16, 17, and 21). Stable Diffusion, on the other hand, awkwardly adds ribbons to subjects to signify the illness: three images showed pink and red ribbons emerging from the skin of a patient, as if it “grew” from their chests (see Fig. 1, images 13 and 14), while another image shows a huge red ribbon covering the face of one of the patients (Fig. 1, image 15), as if defining their whole identity.

Below, we explore how GAI visually engages with the embodied experience of cancer.

### Treatment and impact on the body

Cancer imagery in popular media often omits treatment or embodied consequences, obscuring its medical reality and leading to awareness campaigns that are not always representative of the breadth of cancer experiences—these limitations have been shown to transfer into AI generated images, too [6]. We expand on this by coding five treatment indicators: headbands, hair loss, hospital settings, medical equipment (such as IVs and medical machines) and scars (see Online Resource 5).

In the sample, 102 images (39%) depicted at least one treatment indicator. Headbands appeared in 48% of these (18.7% of the total images), while hair loss was salient in 38.2% (14.9% overall). Hospital settings were less common (13.7% of images with an indicator; 5.3% overall), as was medical equipment (22.2%; 8.8% overall). Cancer scars appeared in four images only, and we found no images were showing postmastectomy tattoos.

Prompts that referred to *people* who have experienced cancer—using terms like “patient,” “person,” or “survivor”—led to more frequent depictions of treatment. For example, 98.2% of “patient” images and 51.8% of “survivor” images included visible signs of treatment, such as IV lines, hospital gowns, or bedridden subjects (see Fig. 1, images 22, 24, 25, and 26). In contrast, prompts using the word “awareness” produced fewer medical details, favoring symbolic or stylized representations over clinical ones.

Hospital settings were also more common in “patient” images: 13 out of the 14 images with hospital settings came from “patient” prompts. These prompts also produced most images where medical equipment was visible (60% of 23). For breast cancer, the connection to medical experiences was more nuanced: 28.7% of breast cancer images showed some form of treatment, but none included hospitals, and only five showed medical equipment (9% of all images from this site). Instead, they favored headbands or hair loss.

Hospitals, gowns, or IVs are particularly visible in Dall-E images (see Fig. 1, images 22 to 27). Stable Diffusion, by contrast, tended to avoid medical equipment but showed a wider emotional range, including patients in pain or crying, often rendered in black and white to evoke a haunting mood (see Fig. 1, images 07 to 12).

These patterns reflect a broader visual trend in cancer communication, particularly within advocacy campaigns and popular media, where treatment is often symbolized rather than shown. Hair loss, for instance, is frequently aestheticized through colorful head coverings, while clinical elements like IV lines or hospital equipment are omitted in favor of more hopeful imagery. The limited presence of medical realism in our dataset suggests that GAI systems reproduce this symbolic visual language unless explicitly prompted otherwise. As a result, the physical and emotional complexities of cancer treatment risk being sanitized in AI-generated content.

In the following section, we explore how GAI visualized people with cancer in more detail.

### Appearance, emotions, and behaviors: how GAIs depict people with cancer

To explore how GAIs imagine people living with and beyond cancer, we coded gender, age, ethnic cues, behaviors, and emotions. The results are organized under two questions: what do people who experience cancer *look* like, and how do they *feel* and *behave*?

#### Appearance: what do people who experience cancer look like?

Our results partly align with the literature: people in AI-generated cancer images are mostly female and White, but not necessarily young (see Online Resource 6).

Building on previous findings that AI-generated images often reflect breast cancer survivorship tropes [6], we found that both GAIs tend to associate cancer with women—particularly when prompts are vague. Of the 178 images where gender was identifiable, 117 (65.7%) depicted only women, 42 (23.6%) only men, and just 19 (10.6%) showed both. Although a few images included androgynous figures or lacked clear gender cues, most were distinctly gendered. Among the 56 images generated using breast cancer-specific prompts, only two showed men. Prompts referencing prostate or pancreatic cancer led to more male representations, but still rarely included both genders in the same image. Across most prompts, women were overrepresented, though those using terms like “awareness” or “patient” produced slightly more gender-diverse results.

In terms of age, our results challenge the common portrayal of cancer in social media and consumer magazines, which frequently feature younger adults. Among the 179 images where life stage was identifiable, most featured adults exclusively, with “patient” prompts more likely to show visible signs of aging. Ethnic diversity, meanwhile, was limited: 72.5% of images depicted only light-skinned individuals, a pattern especially pronounced in breast cancer images. Only 6.0% of all images showed ethnically diverse groups, typically in generic scenes such as rallies. While “patient” prompts produced slightly more diverse results, they still overwhelmingly featured light-skinned individuals (67.2%).

#### Emotion: how do people who experience cancer feel and behave?

Overall, we found two main portrayals of patients: some images showed individuals alone, frowning, or covering their faces (see Fig. 1, images 08, 09, 11, and 12), while others showed cheerful individuals (Fig. 1, images 07 and 10), steeped in brighter colors and often smiling.

Negative emotions were more common with “patient” prompts, showing subjects looking down and frowning. Meanwhile, “person” prompts returned neutral expressions or no faces. Facial expressions were sometimes ambiguous, with elements that did not align (e.g., smiling lips with downcast eyes).

**Fig. 2:**
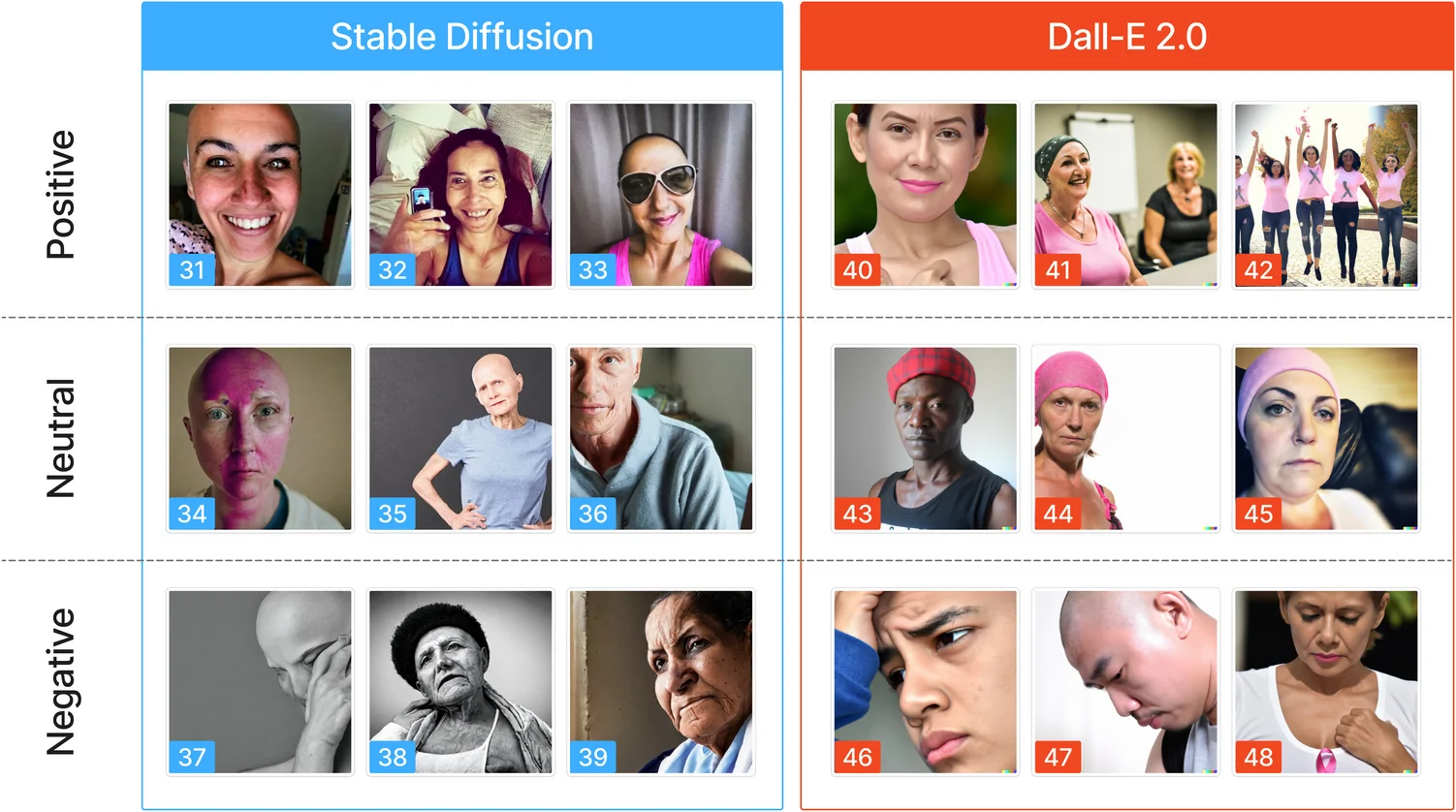
Examples of the emotional tone of cancer images generated by AI. Images were selected to illustrate the results. This figure compares how DALL-E 2.0 (right) and Stable Diffusion (left) visualize emotional states in cancer-related prompts. Images are grouped by emotional tone: positive (e.g., smiling, vibrant colors), neutral (e.g., blank expressions, muted settings), and negative (e.g., sadness, downward gaze, desaturation). The comparison illustrates how generative AI platforms use facial expressions, color, and composition to convey mood

Beyond facial expressions, GAIs rely on body language, color, and props to suggest emotions. In Fig. 2, image 48, the subject places a hand over her chest, visually reinforcing the presence of breast cancer. Desaturation is used in images 37 and 38 to evoke sadness or gravity, while the brighter colors and coordinated pink outfits of image 42 signal optimism and solidarity. In our sample, makeup (shown in 21% of all sample images) often matches clothing, contributing to a stylized, polished appearance, and reflecting ideas of restitution and sorority that are common in cancer advocacy.

Some of these emotional elements can be difficult to interpret. For example, in Fig. 1, image 28, one person faces another who raises a thumb up and a thumb down, as if presenting choices or conveying contrasting attitudes, leaving the emotional tone unresolved.

Notably, many of the images obtained are selfies and solitary portraits: in the sample, 74% of the people were depicted alone. When multiple people were shown, they were often part of a crowd, such as in a rally. Few images showed one-on-one conversations or daily life contexts, as if cancer was a separate reality. This supports Senft Everson et al.’s argument that AI-generated images tend to decontextualize cancer [6].

These visualizations of cancer, survivorship, and people with cancer across GAIs and sets of prompts indicate the potential of GAIs to both perpetuate and challenge problematic discourses, with practical implications for social advocacy that we discuss below.

## Discussion

This is one of the first studies to analyze how Generative AI tools visualize cancer and survivorship. By examining image outputs from Dall-E 2.0 and Stable Diffusion, we demonstrate how GAI reproduces entrenched survivorship discourse while also revealing potential for more inclusive visual representation under specific prompting conditions. These findings extend the work of Senft Everson et al. [6], who identified tonal differences in GAI imagery based on binary prompt terms like “cancer survivor” and “cancer patient.” If AI-generated imagery becomes a staple in the visual language of illness, it may shape not only advocacy design but also public empathy and funding priorities.

Our analysis shows that GAI draws heavily on culturally dominant discourses when visualizing cancer, particularly in breast cancer-related prompts. These tropes appear even when the cancer type is unspecified, underscoring the extent to which breast cancer advocacy has shaped the broader visual vocabulary of cancer. Medical realities such as hospital settings, treatment equipment, and visible bodily consequences were largely absent unless explicitly prompted, suggesting that GAI tools default to philanthropic and commercial-kitsch aesthetics rather than clinical or experiential ones [17, 25].

These patterns raise important concerns about representational bias. AI-generated images were skewed heavily toward White, female, and heteronormative portrayals—especially in the context of breast cancer—while more diverse representations emerged primarily when identity-specific terms like “patient” were included. Such visual imbalances risk reinforcing exclusionary norms that marginalize racialized communities, men with breast cancer, older adults, and people affected by less publicly visible cancers. This not only reflects training data biases but also the risk of flattening complex experiences into aesthetic templates optimized for recognizability or emotional appeal.

The impact of these representational gaps is broader than just aesthetic. In health communication and advocacy, imagery influences public empathy, funding priorities, and how different populations are perceived—or overlooked. If GAI tools are used uncritically in campaigns, social media, or health education, they may inadvertently reproduce exclusionary narratives that hinder the goals of equity-driven survivorship advocacy. This is particularly important as GAI becomes more accessible to influencers, businesses, and institutions that may lack the prompting literacy needed to produce inclusive outputs.

Despite these risks, our results also point to moments where GAI departs from dominant visual tropes. Some images reflect more nuanced emotional tones—from bleak and introspective [61] to joyful and defiant—and occasionally feature non-binary or androgynous subjects, especially when gender cues were not included in the prompt. The absence of militaristic or sports metaphors [12] suggests that GAI is not rigidly bound to older survivorship framings. Further, both platforms showed the capacity to depict medical contexts, diverse bodies, and less curated environments that resemble those found in cancer storytelling online [10]. These instances illustrate the tools’ latent potential: when guided by careful prompting, GAIs can challenge normative imagery and foreground less visible experiences. But this possibility hinges on a level of visual and discursive literacy that cannot be assumed among all users.

These insights carry important ethical implications. AI-generated content may unintentionally promote emotional misrepresentation, aestheticize suffering, or erase certain subjectivities—particularly when it emphasizes positivity and resilience. In turn, this can pressure people with cancer to conform to dominant survivorship scripts, while further obscuring the realities of those who do not “fit” the celebratory narrative. As GAI becomes integrated into public-facing communication, advocacy groups and health institutions must critically assess not only what these tools generate, but how those outputs shape perception, identity, and belonging.

### Limitations

This study offers an exploratory analysis of how generative AI tools visualize cancer and survivorship, but several limitations should be acknowledged. First, our focus on three specific cancer types—breast, prostate, and pancreatic—provided a useful contrast in terms of public visibility and advocacy infrastructure. However, this selection necessarily excludes a broader range of cancers that present different representational challenges. For example, pediatric cancers, blood cancers, or rare cancer types may evoke different visual logics. Future studies could expand the scope to assess how GAI handles less frequently represented conditions, or how it navigates contested spaces.

Second, the study relied on two publicly available text-to-image models: Dall-E 2.0 (now part of ChatGPT) and Stable Diffusion. While these tools are widely used, they represent only a small portion of the GAI ecosystem and reflect specific training data and design choices. As such, our findings may not be transferable to other models, particularly those trained on different image corpora or tuned for clinical contexts. GAI technologies are also continuously evolving; newer versions may respond differently to the same prompts, and current outputs may soon become obsolete. Ongoing comparative research is needed to monitor how representational biases shift—or persist—across model updates and platforms.

Third, we designed prompts to reflect the perspective of a lay user, aiming to simulate how the general public or advocacy groups might engage with GAI tools. While this approach offered insight into accessible outputs, it does not capture how these images are interpreted, appropriated, challenged, or curated by stakeholders such as cancer patients, carers, or advocacy professionals. Future work may include these perspectives through participatory or reception-based research to offer a richer understanding of the social, cultural, and emotional impact of AI-generated imagery.

Finally, this study employed a qualitative, interpretive approach, focusing on visual patterns, tropes, and connotations. While this allowed us to attend to nuance and context, it did not include statistical analysis of image features or formal validation across larger datasets. Future work could combine visual semiotics with computational image analysis or survey-based methods to triangulate findings and assess their broader applicability.

### Practical implications and future research

Our findings indicate that inclusive and ethical use of GAI in cancer communication requires both practitioner-oriented support and further technical investigation.

For health practitioners—particularly those without expertise in AI or socio-cultural representations of cancer—there is a need for accessible tools that guide prompt creation and reduce the risk of defaulting to narrow or stereotypical imagery. Cancer advocacy organizations could develop prompt libraries (i.e., “how to” online resources that guide users wishing to generate images with AI) tailored to different needs (e.g., diagnosis stages, age groups, racial diversity), supported by short, visually guided training and interactive examples. These materials would help democratize the use of GAI while promoting more inclusive and respectful visual communication.

For data scientists, AI researchers, and developers, the next step involves stress-testing these systems through adversarial prompting—deliberately crafting inputs that challenge model defaults and reveal where bias or failure persists. This would help clarify the boundaries of model responsiveness and expose where improvements in prompting still fall short. In parallel, efforts to diversify training datasets—through interdisciplinary collaboration with cancer survivors, carers, practitioners, and visual researchers—will be essential to developing more representative visual vocabularies of illness and survivorship.

At the same time, we find that there is a need for a systematic understanding of how GAI models reproduce visual bias. Future research might begin by identifying the axes along which discriminatory visual discourses of cancer most commonly emerge—for example, through the repetition of normative Whiteness, the erasure of aging or disability, or the flattening of emotional complexity into cheerfulness or heroism. Mapping these visual logics could offer a framework for GAI evaluation and prompt design and serve as the basis for a more comprehensive critical taxonomy of bias in synthetic medical imagery. Existing initiatives, such as the American Cancer Society’s Cancer Action Network resources [62], provide useful precedents for this kind of applied engagement.

Finally, establishing ethical standards for the use of GAI in public health and advocacy campaigns could reduce the risk of reinforcing exclusionary narratives or aestheticizing suffering. Transdisciplinary collaboration among social scientists, medical researchers, advocacy groups, and GAI developers will be critical to ensure these technologies support, rather than undermine, inclusive communication. Such partnerships are essential not only to continue the representational work of past advocacy movements but also to adapt it to the emerging affordances and challenges of generative technologies.

## Conclusion

Using GAI to generate cancer-related visual content offers a valuable way to quickly illustrate the lived experience of cancer without disclosing private information—a clear benefit for researchers, social advocacy, and public health campaigns. But what does GAI reveal about the social imagination of cancer and survivorship, and how might this affect people’s identity and sense of wellbeing?

Our results show that GAIs reflect existing dynamics and gaps in cancer communication. When prompts are unspecific, they default to survivorship and breast cancer signifiers, revealing how deeply these are embedded in public perception. To generate more inclusive images, we needed to use terms like “patient” or “person with cancer.” This lack of diversity risks excluding those whose experiences do not align with dominant survivorship discourse or indeed those who have died with cancer, echoing the same presentational biases denounced since the 1970 s. Such biases could undo decades of advocacy progress by pressuring people with cancer and their carers to conform to a narrative that may not reflect their experiences—doing so more quickly and forcefully than ever. GAIs may also further skew advocacy and research toward more visible and relatable types of cancer, amplifying emotions such as compassion and identification and marginalizing stigmatized cancer experiences [63].

Despite these challenges, GAIs also have the potential to produce more inclusive visual material. The key lies in how we “speak” to GAIs and how prompts are crafted. Stepping outside normative survivorship discourses requires a nuanced understanding of prompt-writing and the complexities of cancer communication. While advocacy groups and organizations may possess this expertise, lay users—such as businesses, influencers, and celebrities—cannot be expected to have the same depth of knowledge. When they simply want to create an image to show solidarity, for instance on World Cancer Day, they may rely on generic or standardized outputs. Such outputs may perpetuate stereotypes.

These findings can directly inform the design of advocacy materials and public health campaigns by highlighting how prompt language shapes representational outcomes. Addressing these representational limitations will require sustained collaboration between researchers, advocacy groups, and developers. Researchers can map patterns of bias and exclusion, while advocates contribute grounded knowledge of lived experience and representational needs. Developers, in turn, play a critical role in adjusting model architecture, training datasets, and user interfaces to reduce harm. This trans-disciplinary collaboration is necessary to ensure that generative AI tools evolve in ways that support inclusive, ethical cancer communication.

## Supplementary Information

Below is the link to the electronic supplementary material.

Supplementary file1 (XLSX 11 KB)

Supplementary file2 (XLSX 11 KB)

Supplementary file3 (XLSX 10 KB)

Supplementary file4 (XLSX 13 KB)

Supplementary file5 (XLSX 12 KB)

Supplementary file6 (XLSX 14 KB)

## Data Availability

Supplemental tables are provided as online resources. All images generated are freely available upon request to the corresponding author.

## Footnotes

1 Dalle-E 2.0 is developed by OpenAI, which also produces ChatGPT, while Stable Diffusion is built by LMU’s Machine Vision & Learning group [1].

2 At the time of writing, Dall-E was a standalone product. It has since been incorporated into ChatGPT.

3 Note that different signifiers can appear together in the same image.
